# A bioluminescence reporter mouse that monitors expression of constitutively active β-catenin

**DOI:** 10.1371/journal.pone.0173014

**Published:** 2017-03-02

**Authors:** Maria M. Szwarc, Ramakrishna Kommagani, Mary C. Peavey, Lan Hai, David M. Lonard, John P. Lydon

**Affiliations:** 1 Department of Molecular and Cellular Biology, Baylor College of Medicine, Houston, TX, United States of America; 2 Department of Obstetrics & Gynecology, Washington University School of Medicine, St. Louis, MO, United States of America; 3 Department of Obstetrics and Gynecology, Baylor College of Medicine, Houston, TX, United States of America; University of Tennessee Health Science Center, UNITED STATES

## Abstract

This short technical report describes the generation and characterization of a bioluminescence reporter mouse that is engineered to detect and longitudinally monitor the expression of doxycycline-induced constitutively active β-catenin. The new responder transgenic mouse contains the TetO-ΔN89β-Cat^TMILA^ transgene, which consists of the tet-operator followed by a bicistronic sequence encoding a stabilized form of active β-catenin (ΔN89β-catenin), an internal ribosome entry site, and the firefly luciferase gene. To confirm that the transgene operates as designed, TetO-ΔN89β-Cat^TMILA^ transgenic mouse lines were crossed with an effector mouse that harbors the mouse mammary tumor virus-reverse tetracycline transactivator (MMTV-rtTA) transgene (termed MTB hereon), which primarily targets rtTA expression to the mammary epithelium. Following doxycycline administration, the resultant MTB/Cat^TMILA^ bigenic reporter exhibited precocious lobuloalveologenesis, ductal hyperplasia, and mammary adenocarcinomas, which were visualized and monitored by *in vivo* bioluminescence detection. Therefore, we predict that the TetO-ΔN89β-Cat^TMILA^ transgenic responder mouse—when crossed with the appropriate effector transgenic—will have wide-applicability to non-invasively monitor the influence of constitutively active β-catenin expression on cell-fate specification, proliferation, differentiation, and neoplastic transformation in a broad spectrum of target tissues.

## Introduction

Apart from comprising stable cell adheren junctions, β-catenin is a critical effector of the Wnt signaling pathway [1–3]. In Wnt’s absence, cytoplasmic β-catenin is normally targeted for ubiquitin-mediated proteolysis by glycogen synthase kinase-3β (GSK-3β) within a destruction-complex with adenomatous polyposis coli (APC) and axin [4]. Specifically, GSK-3β phosphorylates conserved N-terminal serine and threonine residues of β-catenin that post-translationally mark β-catenin for destruction [5, 6]. In Wnt’s presence, GSK-3β is inactivated thereby allowing β-catenin stabilization and nuclear translocation [7]. Within the nucleus, β-catenin complexes with members of the T-cell factor/lymphoid enhancer factor (Tcf/Lef) family of transcription factors that together control the expression of a myriad of target genes involved in normal tissue development and function (http://www.stanford.edu/~rnusse/pathways/targets.html).

Not surprisingly, aberrant β-catenin signaling is causal for numerous embryonic and postnatal developmental abnormalities—including tumorigenesis—in diverse anatomic sites [1]. Mutations that prevent phosphorylation and turnover of the cytoplasmic pool of β-catenin lead to the accumulation of a constitutively active form of β-catenin that can inappropriately induce downstream Wnt target genes in a Wnt-independent manner. Importantly, the genetically engineered mouse was instrumental in validating many of the above findings in an *in vivo* context.

Because the engineered mouse has been a pivotal *in vivo* experimental model to study Wnt signaling in general and β-catenin signaling in particular, it is imperative that the full potential of the mouse is attained to further advance our understanding of β-catenin action *in vivo*. Accordingly, this brief technical report describes the generation and first-line characterization studies of a new TetO-ΔN89β-Cat^TMILA^ transgenic mouse that enables noninvasive *in vivo* detection of constitutively active β-catenin expression using a bioluminescence reporter. Given the importance of β-catenin signaling in development, adult tissue homeostasis, and tumorigenesis, we believe this bioluminescence reporter mouse will have wide-applicability.

## Materials and methods

### Creation of the TetO- ΔN89β-Cat^TMILA^ transgenic mouse

Mice were housed in an AAALAC accredited *vivarium* at Baylor College of Medicine, which operates a 12h-light: 12h-dark recurrent photocycle in temperature-controlled mouse rooms (22 ± 2°C). Mice were fed irradiated Teklad global soy protein-free extruded rodent diet (Harlan Laboratories, Inc., Indianapolis, IN) and fresh water *ad libitum* when not treated with doxycycline (see below). The TetO- ΔN89β-Cat^TMILA^ transgene was generated by inserting a cDNA (2.1kb) encoding *Xenopus* ΔN89β-catenin [8–10] into unique EcoR1/Cla1 restriction sites in the TMILA reporter vector (7.4kb (Chodosh plasmid #652)) [11]. The 89 amino acid N-terminal deletion in ΔN89β-catenin renders the truncation mutant constitutively active [12, 13]. The insertion of the ΔN89β-catenin cDNA into the TMILA reporter vector positions the ΔN89β-catenin cDNA downstream of the cytomegalovirus (CMV) minimal promoter and a tandem repeat of seven Tet operator (TetO) sequences derived from the pTetSplice vector [14]. With this cloning approach, the ΔN89β-catenin cDNA insertion is sequentially followed by an internal ribosome entry site (IRES) and the codon optimized firefly luciferase 2 gene cassette from *Photinus pyralis* (Promega, Madison, USA). A strong Simian virus 40 (SV40) splicing/polyadenylation sequence is included at the 3’ end of the transgene. The resultant TetO-ΔN89β-Cat^TMILA^ transgene (6.6kb) was released from pTetSplice vector sequences (2.9kb) with Not1 digestion, isolated from vector sequences, and then purified for microinjection into pronuclei of single-cell embryos of the FVB/N inbred strain. Founder mice (F0) and their progeny were identified by PCR genotyping of genomic DNA isolated from tail snips. The PCR primers that were used to detect the TetO- ΔN89β-Cat^TMILA^ transgene were previously described [10]. Each of the four TetO- ΔN89β-Cat^TMILA^ responder transgenic lines (F1) was crossed with the MTB effector transgenic [14] to generate MTB/Cat^TMILA^ bigenics that were maintained in the FVB/N background strain. All studies described herein were conducted with nulliparous mice that were hemizygous for the transgene.

### Ethics statement

All mouse studies were conducted in accordance with the Guide for the Care and Use of Laboratory Animals published by the National Institutes of Health and animal protocols approved by the Institutional Animal Care and Use Committee (IACUC) of Baylor College of Medicine under protocol numbers AN-1513 and AN-544. Euthanasia was conducted by cervical disarticulation while under surgical plane of anesthesia. CO2 euthanasia was conducted by use of automated CO2 euthanasia chambers (Euthanex, Palmer, PA).

### Transgene induction by doxycycline

Responder transgene expression in four-week-old MTB/Cat^TMILA^ bigenics was induced by feeding rodent chow meal supplemented with doxycycline (6g/kg (BioServ, St. Louis, MO)). Dispensed in light protected bottles, rodent water contained doxycycline (2mg/ml (BD Clontech, San Diego, CA)) along with 5% sucrose to ameliorate taste aversion [10, 15, 16]. To maintain continued induction efficacy, doxycycline supplemented water was replaced every third day.

### Bioluminescence imaging

Twenty four hours prior to mouse imaging, the ventral area of the skin was depilated using a commercially available depilating cream. On the day of imaging, isoflurane-anesthetized mice were intraperitoneally injected with RediJect D-luciferin bioluminescent substrate (PerkinElmer, Waltham, MA (150mg/kg)) in sterile 0.9% saline. After 5 minutes, bioluminescence was detected and recorded using the Bruker FX Pro Imager (Bruker, Billerica, MA) equipped with an isoflurane manifold for continuous anesthesia; mice were placed in the ventral recumbent position for bioluminescence detection. Bioluminescence was captured within a 30 second exposure time with 4x4 pixels binning followed by X-ray image capture (10 second exposure). Using Bruker Molecular Imaging software (v.7.1.3.20550), bioluminescence images were exported in pseudo color format with matched rainbow-colored bar scales (minimum and maximum photons/second). For final presentation purposes, bioluminescence images were overlaid upon the corresponding grey-scale X-ray image.

### Whole mount, immunohistochemical, and western immunoblot analysis

Carmine-red stained mammary gland whole-mounts were performed as previously reported [10, 15, 16]. The antibodies and conditions used for immunohistochemical detection of the myc-epitope tag and 5’-bromo-2’-deoxyuridine (BrdU) incorporation have been described [10]. To determine the percentage of mammary epithelial cells that is immunopositive for BrdU incorporation, 6 control monogenic and 5 MTB/Cat^TMILA^ bigenics were used. Note: ductal and alveolar epithelial cells were counted for the MTB/Cat^TMILA^ bigenic whereas only ductal epithelial cells could be counted for the control monogenic gland since there are very few alveolar cells in the adult virgin mammary epithelium to get an equivalent count. Only intensely stained (dark brown) nuclei for BrdU incorporation were included in the cell count. The average number of immunopositive cells was calculated from a total of 500 mammary epithelial cells from three separate mammary gland sections per mouse. Final counts were expressed as an average percentage mean of cells counted. Antibodies and conditions used for western immunoblot detection of β-catenin, the myc-epitope tag, cyclin D1, and β-actin in protein isolates from mammary epithelial cells and tumor tissue have been previously detailed [10]. Mammary epithelial cell isolation was followed according to established methods [17, 18].

### Mammary tumor induction

Mice chronically administered doxycycline for mammary tumor induction were checked twice weekly by manual palpation. Mice were euthanized when mammary tumors reached approximately 1.0cm in diameter as measured by Vernier calipers. For each mouse, tumor size, number, and ventral location were recorded prior to euthanasia. GraphPad Prism 6 software (GraphPad Software, Inc., La Jolla, CA) was used to generate and statistically analyze tumor-free Kaplan-Meier plot. The two-sided log-rank test was used to determine significance of the difference in tumor-free rate between virgin bigenic mice that chronically received food and water supplemented with doxycycline (n = 21) and bigenic mice maintained on regular mouse chow (n = 28); a p-value <0.05 was considered significant.

## Results and discussion

### Design and generation of the TetO- ΔN89β-Cat^TMILA^ responder transgenic mouse

**Fig 1:**
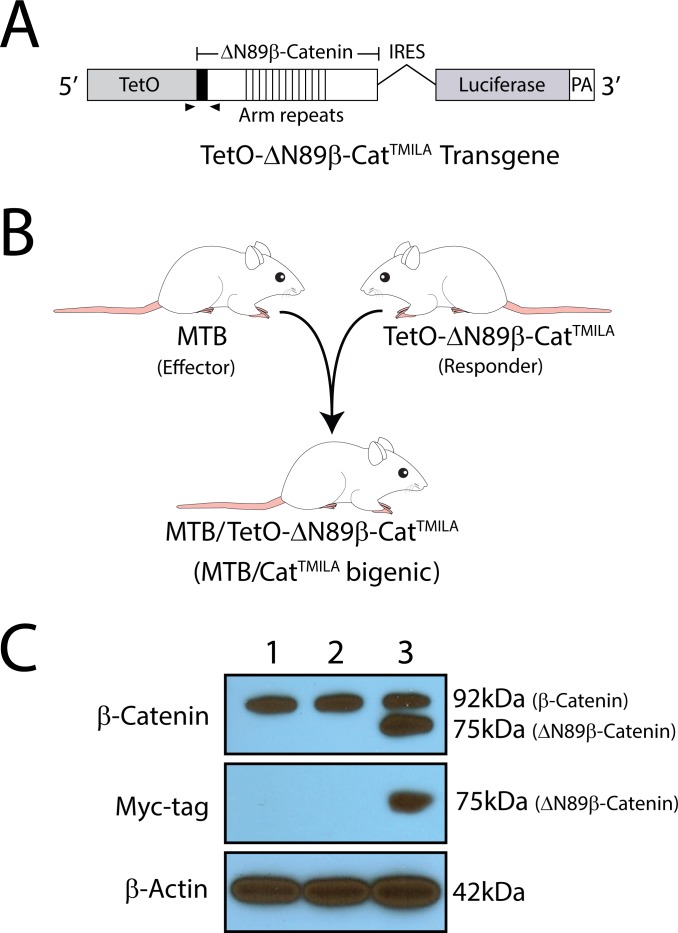
Generation of the MTB/Cat^TMILA^ bigenic mouse. (A) Design of the TetO- ΔN89β-Cat^TMILA^ transgene. The ΔN89β-catenin cDNA (2.1kb) was cloned into a single EcoR1 restriction site downstream of the TetO sequence in the TMILA (7.4kb) cloning vector [11]. The ΔN89β-catenin cDNA encodes the truncated *Xenopus* β-catenin protein with a myc-epitope tag fused in-frame at its N-terminus (black box). The location of the PCR primers for genotyping (black arrowheads) as well as the 13 centrally located Armadillo repeats (Arm repeats) is indicated. The inserted ΔN89β-catenin cDNA is followed by an IRES and a cDNA encoding the firefly luciferase protein. A SV40 polyadenylation signal (PA) serves as a strong transcriptional termination signal. The TetO- ΔN89β-Cat^TMILA^ transgene was linearized with Not1, isolated from vector sequences, and purified prior to pronuclear microinjection. (B) Schematic depicts the breeding strategy to generate the MTB/Cat^TMILA^ bigenic mice by crossing the MTB effector transgenic [14] with TetO-ΔN89β-Cat^TMILA^ responder transgenic. (C) Typical western immunoblot of isolated mammary epithelial cell protein. Lane 1, 2, and 3 denote mammary epithelial protein isolated from wild type ((WT) or non-transgenic) control (without doxycycline), MTB/Cat^TMILA^ bigenic (without doxycycline), and MTB/Cat^TMILA^ bigenic mice on food and water supplemented with doxycycline for 1-month respectively. Using antibodies to full-length β-catenin and the myc-epitope tag, the transgene-derived ΔN89β-catenin protein (75kDa) is only detected in the MTB/Cat^TMILA^ bigenic treated with doxycycline (lane 3); β-actin serves as a loading control. Each lane represents a protein isolate pooled from four individual mice per genotype and treatment.

The design of the TetO-ΔN89β-Cat^TMILA^ transgene is schematically shown in **Fig 1A**. The *Xenopus* ΔN89β-catenin cDNA (2.1kb) was inserted between EcoR1 (5’) and Cla1 (3’) restriction sites in the multiple cloning cassette of the TMILA vector (7.4kb) [11]. Note: the *Xenopus* and human β-catenin protein sequences share 98% homology. To enable specific immunodetection, the ΔN89β-catenin cDNA was engineered to express in-frame a myc-epitope tag at the N-terminus of the ΔN89β-catenin protein [10]. Due to deletion of its first 89 amino acids, the ΔN89β-catenin protein is constitutively active. The insertion of the ΔN89β-catenin cDNA into the TMILA vector positions the cDNA downstream of the Tet operator (TetO) sequences and upstream of (in sequential order) the IRES, luciferase reporter, and the SV40 intron/polyA cassette. With standard transgenic methodology [10, 15, 16, 19], four out of six mice positive for the TetO- ΔN89β-Cat^TMILA^ transgene transmitted the transgene through the germline (#G4704; #G4715; #G4717; and #G4720). To confirm that ΔN89β-catenin is expressed by these transgenics in response to doxycycline administration, each of the four transgenic lines was crossed with the MTB effector transgenic [14] to generate the MTB/Cat^TMILA^ bigenic (**Fig 1B**). Unless otherwise stated, data derived from the #G4715 are shown herein which are representative of the other transgenic lines. Western immunodetection for β-catenin and the myc-epitope tag demonstrates that the transgene-derived ΔN89β-catenin protein (75kDa) is specifically expressed in mammary epithelial protein isolates derived from the MTB/Cat^TMILA^ bigenic in response to doxycycline administration (**Fig 1C**).

### Doxycycline induction of ΔN89β-catenin expression in the mammary epithelium of the virgin MTB/Cat^TMILA^ bigenic is detected by bioluminescence

**Fig 2:**
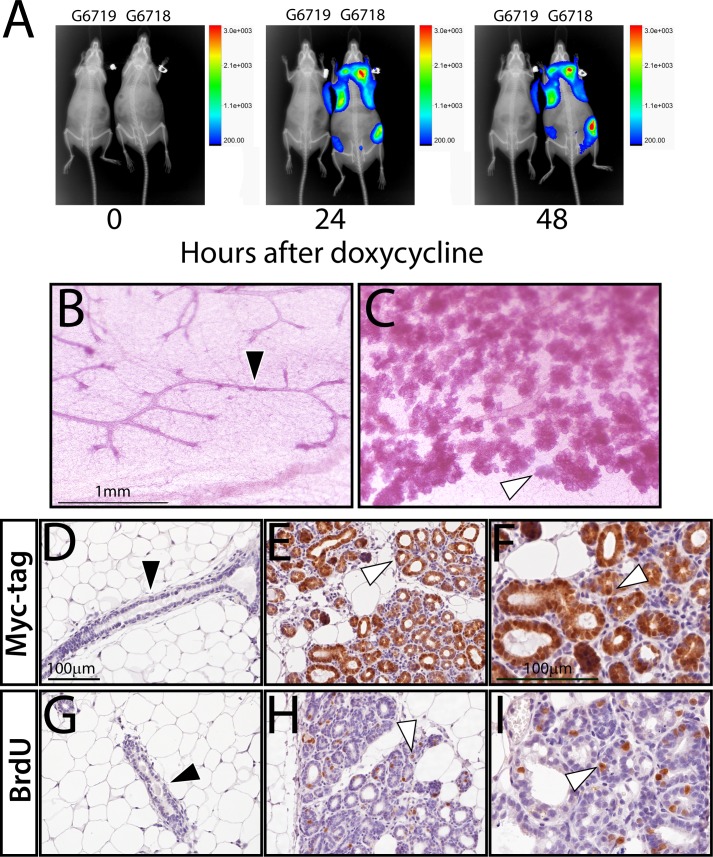
Doxycycline-induced bioluminescence in the mammary gland of the MTB/Cat^TMILA^ bigenic. (A) Overlay of full-body bioluminescence and x-ray images of monogenic control (G6719 (ear tag#)) and MTB/Cat^TMILA^ bigenic (G6718 (ear tag#)) mice following 0, 24, and 48 hours of doxycycline intake. By 24 hours of doxycycline administration, bioluminescence activity is detected in the #2, #3 (thoracic), and #4 (inguinal) mammary glands of the MTB/Cat^TMILA^ bigenic (G6718) but not in the monogenic control mouse (G6719). (B) Whole mount of mammary gland from monogenic control mouse (G6719) shows normal ductal morphogenesis (black arrowhead) following 1-week of doxycycline administration. (C) Mammary gland whole mount analysis shows precocious lobuloalveologenesis (white arrowhead) in the similarly treated MTB/Cat^TMILA^ bigenic (G6718). Scale bar in (B) applies to (C); see S1 Fig for corresponding low magnification images. (D) Myc-epitope tag immunohistochemistry does not detect myc-epitope tagged ΔN89β-catenin expression in the mammary epithelium of the doxycycline treated monogenic (G6719) control (black arrowhead). (E) Myc-tagged ΔN89β-catenin expression is clearly detected in the mammary epithelium of the similarly treated MTB/Cat^TMILA^ bigenic (white arrowhead); (F) is a higher magnification of (E). (G) Image shows a representative transverse section of an epithelial duct in the mammary gland of the doxycycline-treated monogenic (G6719) control mouse, which scores negative for BrdU incorporation following BrdU immunohistochemistry (black arrowhead). (H) Numerous cells scoring positive for BrdU incorporation are detected in the mammary epithelium of the similarly treated MTB/Cat^TMILA^ bigenic (white arrowhead); (I) is a higher magnification. Scale bar in (D) and (F) apply to (E, G, and H) and (I) respectively. See S1 Fig for more details and quantitation of BrdU positive cells in the mammary epithelium of both genotypes following doxycycline administration.

Using the luciferase reporter as a surrogate for transgene-derived ΔN89β-catenin expression, the bioluminescence emission signal was detected as early as 24 hours following doxycycline administration in the MTB/Cat^TMILA^ bigenic (**Fig 2A**). The bioluminescence signal was detected in the majority of mammary glands (thoracic and inguinal) in the adult virgin MTB/Cat^TMILA^ bigenic; as expected, the bioluminescence signal was not detected in the control mouse (**Fig 2A**). Within a short time-period, only the mammary gland of the doxycycline-treated MTB/Cat^TMILA^ bigenic exhibited precocious lobuloalveologenesis and ductal side-branching (**Fig 2B** and **2C**). This result demonstrates that the TetO-ΔN89β-Cat^TMILA^ responder transgene both operates as a bioluminescent reporter and causes β-catenin-dependent disruption of epithelial growth homeostasis. Immunohistochemical staining for the myc-epitope tag and BrdU incorporation demonstrated that ΔN89β-catenin expression was restricted to the nucleus and cytoplasm of the mammary epithelium and that this expression pattern was coincident with mammary epithelial hyperplasia (**Fig 2D–2I**). Collectively, these data show that bioluminescence detection can forecast ΔN89β-catenin induced mammary epithelial abnormalities in the MTB/Cat^TMILA^ bigenic reporter mouse.

### Longitudinal monitoring of mammary tumor progression in the MTB/Cat^TMILA^ bigenic reporter mouse

**Fig 3:**
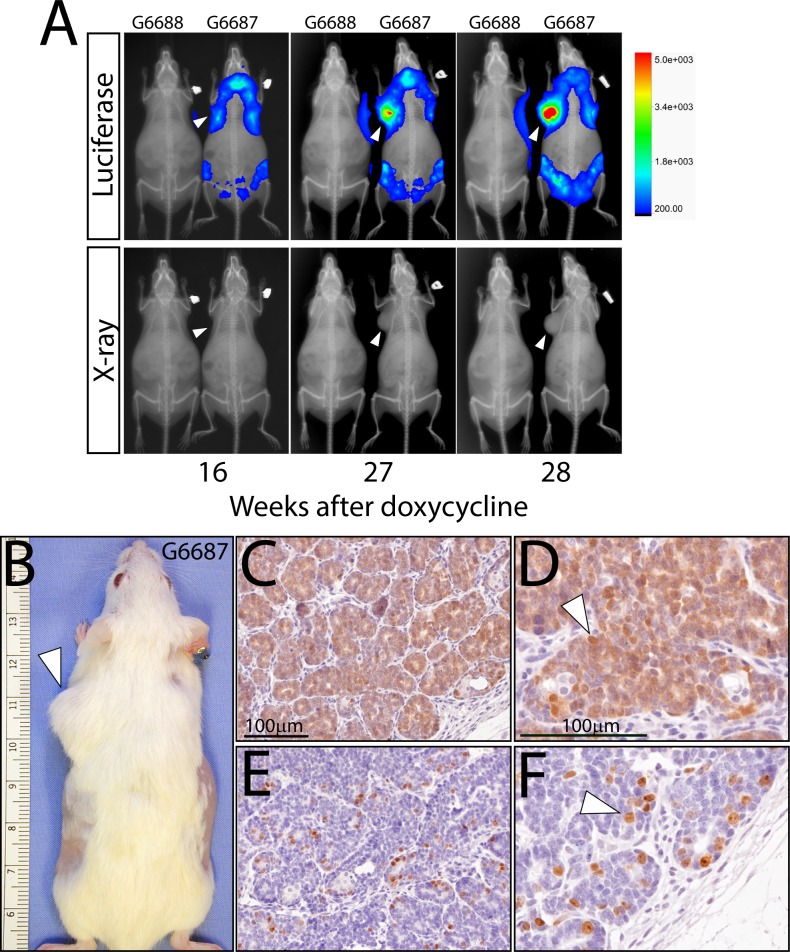
Bioluminescence detection of the emergence of doxycycline-induced mammary tumors in the MTB/Cat^TMILA^ bigenic. (A) Overlay of whole-body bioluminescence and X-ray images shows mammary tumor enlargement in the MTB/Cat^TMILA^ bigenic (G6687) following doxycycline administration for the time periods indicated. Note the emergence of a thoracic mammary tumor (white arrowhead) in the bigenic. As expected, luciferase activity is not detected in the similarly treated monogenic control (G6688). Corresponding whole-body X-rays are shown alone in the bottom panels to enable clear visualization of the mammary tumor mass (white arrow head). (B) The MTB/Cat^TMILA^ bigenic (G6687) is shown following bioluminescence monitoring; note the thoracic mammary tumor detected in (A) above (white arrowhead). (C) and (D) are low and high magnification images respectively of mammary tumor tissue sections immunostained for myc-tagged ΔN89β-catenin expression; note: that most tumor cells score positive for myc-tag immunoreactivity (white arrowhead). (E) and (F) are low and high magnification images of mammary tumor tissue sections stained for BrdU incorporation; many tumor cells are immunopositive for BrdU incorporation (white arrowhead). Scale bar in (C) and (D) applies to (E) and (F) respectively.

**Fig 4:**
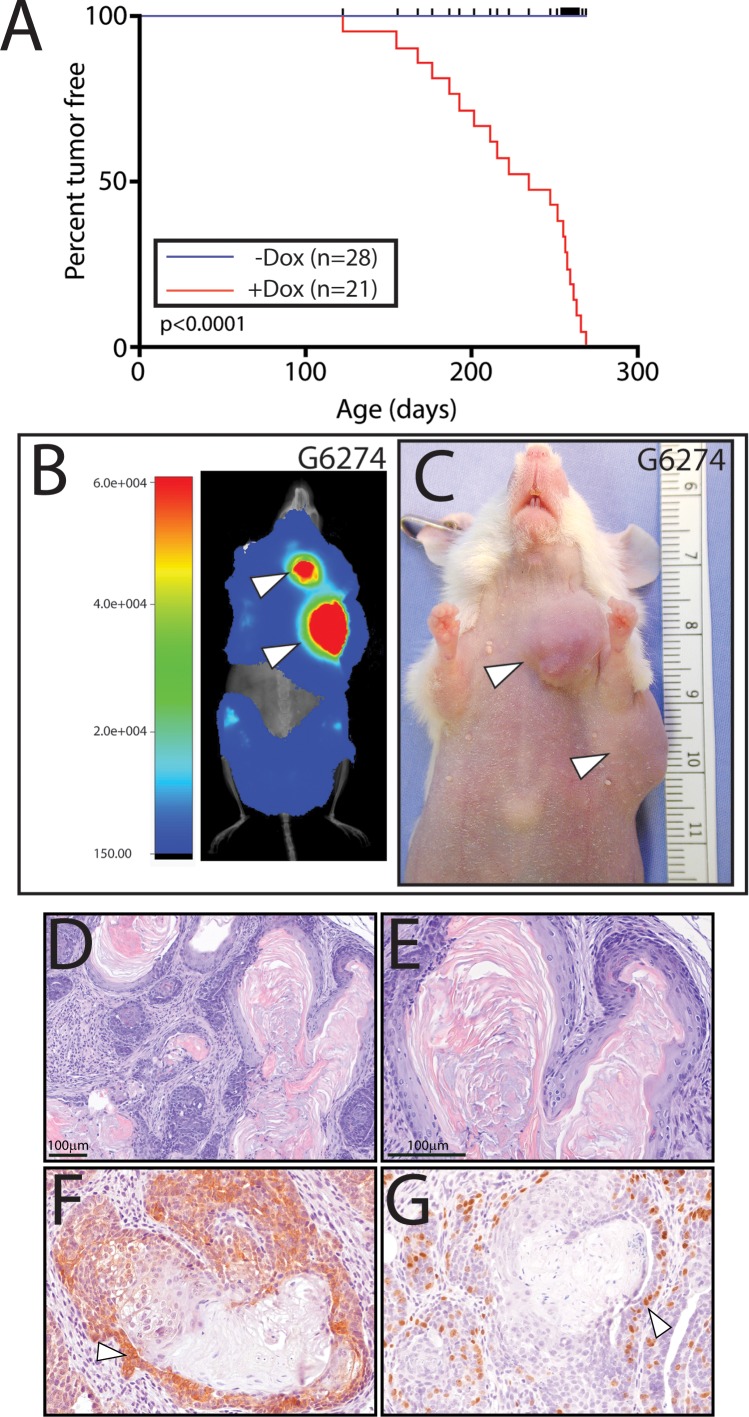
Complete penetrance of the mammary tumor phenotype in the MTB/Cat^TMILA^ bigenic reporter. (A) Kaplan-Meier tumor free plot for MTB/Cat^TMILA^ bigenics without doxycycline administration (n = 28 (blue)) and doxycycline-treated MTB/Cat^TMILA^ bigenics (n = 21 (red)) showing percent tumor free on the Y-axis *versus* age (in days) on the X-axis. (B) Overlay of whole-body bioluminescence and x-ray images of a MTB/Cat^TMILA^ (G6274) bigenic revealing two ipsilateral mammary gland tumors (#2 and #3 thoracic mammary glands (white arrowheads)). (C) The MTB/Cat^TMILA^ (G6274) bigenic reporter exhibiting two mammary tumors shown in (B (white arrowheads)). (D) Hematoxylin and eosin staining reveals that a subset of MTB/Cat^TMILA^ mammary tumors exhibit histologic characteristics consistent with squamous differentiation as evidence by the presence of pilar-like structures of confluent swirls of laminar keratin [20, 21]; (E) higher magnification of image shown in (D). (F) Typical immunostaining for myc-tagged ΔN89β-catenin expression in these tumors (white arrowhead). (G) Representative staining for BrdU incorporation in these tumors. Note that BrdU positive cells are localized to the region of the tumor that expresses ΔN89β-catenin (compare F with G (white arrowheads)); scale bar in (D) and (E) corresponds to (F) and (G) respectively.

With continuous doxycycline administration, the MTB/Cat^TMILA^ bigenic reporter mouse develops palpable mammary tumors that can be detected and longitudinally monitored by bioluminescence (**Fig 3A**). As expected these mammary tumors are mostly adenocarcinomas that exhibit a strong immunopositive signal for the transgene-derived myc-epitope tag and are highly proliferative (**Fig 3B–3F**). The mammary tumor phenotype is 100% penetrant and is completely dependent on doxycycline administration (**Fig 4A**); many of the MTB/Cat^TMILA^ bigenic reporters exhibit multifocal mammary tumors that are readily visualized by bioluminescence detection (**Fig 4B** and **4C**). Although most of the MTB/Cat^TMILA^ mammary tumors are adenocarcinomas (**Fig 3B–3F**), approximately 15% of tumors are squamous metaplasias [20, 21] that are strongly immunopositive for the transgene-derived myc-epitope tag and BrdU incorporation (**Fig 4D–4G**). Interestingly, approximately 10% of MTB/Cat^TMILA^ palpable mammary tumors do not regress following doxycycline withdrawal (S2A Fig). Bioluminescence monitoring clearly reveals that transgene expression activity is rapidly attenuated following doxycycline withdrawal but mammary tumor volume does not decrease (S2A and S2B Fig). These findings are confirmed by western blot and immunohistochemical analysis which show absence of ΔN89β-catenin protein in mammary tumors that continue to expand despite the absence of doxycycline exposure (S2C and S2D Fig**)**. For the majority of mice, however, mammary tumors regress within 2-weeks following doxycycline withdrawal (S3 Fig).

### Doxycycline-induction of ΔN89β-catenin expression in the murine salivary gland can be temporally monitored using bioluminescence detection

**Fig 5:**
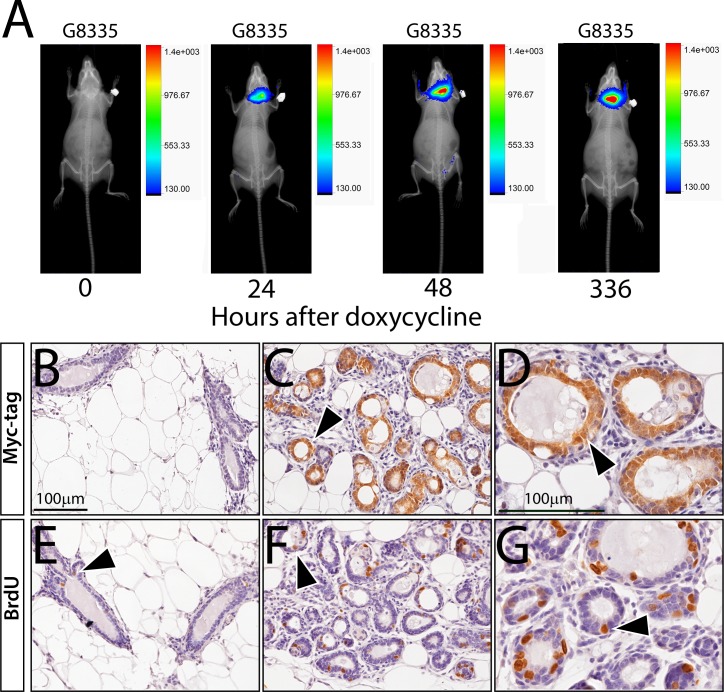
Bioluminescence monitoring of doxycycline-induced ΔN89β-catenin expression in the salivary gland of the MTB/Cat^TMILA^ bigenic. (A) Overlay of whole body bioluminescence and X-ray images of MTB/Cat^TMILA^ bigenic mouse following doxycycline administration for the indicated time periods. Note the localization of the bioluminescence signal to the submandibular salivary gland of the MTB/Cat^TMILA^ mouse (G8335). (B-D) and (E-G) panels represent salivary gland tissue immunostained for the transgene-derived myc-tagged ΔN89β-catenin protein and BrdU incorporation respectively. (B) Salivary gland tissue from doxycycline-treated monogenic control does not score positive for myc-tagged ΔN89β-catenin expression. (C) Cystic hyperplasia with strong immunostaining for myc-tagged ΔN89β-catenin expression is evident in salivary gland tissue isolated from the MTB/Cat^TMILA^ bigenic following 336 hours of doxycycline administration (black arrowhead). (D) is a higher magnification image of (C). (E) Salivary gland epithelial cells positive for BrdU are not evident in salivary gland tissue derived from doxycycline-treated monogenic control mice (black arrowhead). (F) Many cells score positive for BrdU incorporation in salivary gland tissue isolated from the similarly treated MTB/Cat^TMILA^ bigenic (black arrowhead); a higher magnification is shown in (G (black arrowhead)). Note: To date, we have not detected palable salivary tumors in these mice. Scale bar in (B) and (D) apply to (C, E, and F) and (G) respectively.

Bioluminescence monitoring also reveals that progeny from one transgenic line (#G4704) exhibit particularly strong ΔN89β-catenin expression in the submandibular salivary gland with low expression in the mammary epithelium (**Fig 5A**). Following chronic doxycycline exposure, histological analysis of all bigenic mice from this transgenic line revealed that the salivary gland exhibited epithelial hyperplasia that was strongly positive for myc-epitope tag expression (**Fig 5B–5G**). This result is not surprising as the MTB effector mouse has been shown to express rtTA activity in the salivary gland [14], an exocrine tissue developmentally similar to the mammary gland. However, we did not observe strong transgene expression in both the salivary and mammary gland in any transgenics examined. Because dysregulation of β-catenin signaling has been shown to elicit salivary gland tumorigenesis [22–24], this model may be useful as a non-invasive tool to explore further the involvement of β-catenin signaling in the ontogenesis of this poorly understood head and neck cancer.

## Conclusion

We and others previously generated variations of the TetO- ΔN89β-Catenin transgenic effector mouse that was designed to conditionally induce constitutively activated ΔN89β-catenin protein in response to doxycycline [10, 25]. In this short technical report, we describe an important improvement to this model with the generation of the TetO- ΔN89β-Cat^TMILA^ transgenic, which has the added capability of bioluminescence detection. Because of the obvious advantages of optical bioluminescence imaging—a rapid, sensitive, and user-friendly optical modality for temporal assessment of tumor progression and therapy—along with the pleiotropic role of β-catenin signaling in normal and abnormal tissue homeostasis, we anticipate that this new bioluminescence reporter mouse will prove to be invaluable for future investigations to discern the normal and pathogenic role of β-catenin *in vivo*.

## Supporting information

S1 Fig Doxycycline-induction of precocious lobuloalveologenesis in the mammary gland of the MTB/Cat^TMILA^ reporter mouse. (A) and (B) represent low magnification whole mount images of mammary gland tissue isolated from a monogenic control (G6719) and a MTB/Cat^TMILA^ bigenic (G6718) respectively following doxycycline administration for 1-week (see Fig 2). Scale bar in (A) applies to (B); LN denotes lymph node in inguinal mammary gland. At increasing magnification, (C-E) show a typical transverse section of a doxycycline-treated control monogenic mammary duct with a single luminal epithelial cell scoring positive for BrdU incorporation (white arrowhead). At increasing magnification, (F-H) show numerous alveolar epithelial cells in the doxycycline-treated MTB/Cat^TMILA^ bigenic mammary gland that are immunopositive for BrdU incorporation (white arrowhead). As a positive control for BrdU immunostaining, (I) shows BrdU positive cells in the lymph node of inguinal (#4) mammary gland of the doxycycline-treated monogenic control (white arrowhead). (J) shows the extensive alveologenesis and cellular proliferation (white arrowhead) that occurs in the doxycycline-treated MTB/Cat^TMILA^ bigenic mammary gland. Scale bar in (C-E) applies to (F-H) respectively; scale bar in (E) also applies to (I-J). (K) displays a histogram of the mean percentage of cells (± s.e.m.) scoring positive for BrdU incorporation in ductal epithelium of the doxycycline-treated control monogenic (n = 6) and in the ductal and alveolar epithelium of the doxycycline-treated MTB/Cat^TMILA^ bigenic sibling (n = 5); ***denotes P value <0.0001.
(TIF)

S2 Fig Subset of MTB/Cat^TMILA^ mammary gland tumors do not regress following doxycycline removal. (A) Overlay of whole body bioluminescence and x-ray images of monogenic control (G6101) and MTB/Cat^TMILA^ (G6100) bigenic following removal of doxycycline for the time period indicated. By 120 hours post doxycycline removal, transgene-derived luciferase activity is significantly attenuated but mammary tumor mass is not reduced (white arrowhead). (B) Top two panels (low and high magnification) show MTB/Cat^TMILA^ (G6100) bigenic at 0h after doxycycline withdrawal; white arrowhead points to thoracic mammary gland tumor shown in (A). Two bottom panels (low and high magnification) show the MTB/Cat^TMILA^ (G6100) bigenic 144 hours following doxycycline withdrawal. Note that the mammary tumor has not decreased in size following de-induction of the transgene (white arrowhead). (C) Western immunoblot of protein isolated from: WT mammary epithelial cells (lane 1), three separate MTB/Cat^TMILA^ bigenic mammary tumors on doxycycline (lanes 2–4), and MTB/Cat^TMILA^ bigenic mammary tumor off doxycycline for 144 days (lane 5). Note the absence of transgene-derived ΔN89β-catenin protein in lane 5. High levels of cyclin D1 protein expression is detected in all mammary tumor samples (lanes 2–5); β-actin serves as a loading control. (D) Immunohistochemistry does not detect transgene-derived myc-tagged ΔN89β-catenin in mammary tumor tissue (adenocarcinoma) derived from MTB/Cat^TMILA^ bigenic that are off doxycycline for 144 hours. The same mammary tumor tissue is highly proliferative as evidenced by numerous tumor cells scoring positive for BrdU incorporation (white arrowhead). Scale bar applies to both panels.
(TIF)

S3 Fig The majority of palpable mammary tumors in the MTB/Cat^TMILA^ bigenic that are induced by doxycycline fully regress by 14 days following doxycycline withdrawal. A representative MTB/Cat^TMILA^ bigenic with a palpable mammary tumor is shown (black arrowhead) in the top two panels (low and high magnification). For this representative mouse, 136 days of doxycycline administration was required to induce a palpable thoracic (#3) mammary tumor with a ~1cm diameter. Following 6-days on a standard diet without doxycycline (or de-induction), the size of the same tumor rapidly reduced (middle two panels). By 12-days without doxycycline in the diet, the thoracic mammary tumor in the MTB/Cat^TMILA^ bigenic is undetectable by manual palpation. Of the MTB/Cat^TMILA^ bigenic mice in this study (n = 20), 18 mice showed rapid mammary tumor regression within 14-days whereas 2 mice did not show mammary tumor regression within this time period (S2 Fig).
(TIF)
