# Supplementary figures and images for: A bioluminescence reporter mouse that monitors expression of constitutively active β-catenin
Source: PLoS One. 2017 Mar 2;12(3):e0173014. doi: 10.1371/journal.pone.0173014 (PMC5333872; doi:10.1371/journal.pone.0173014)

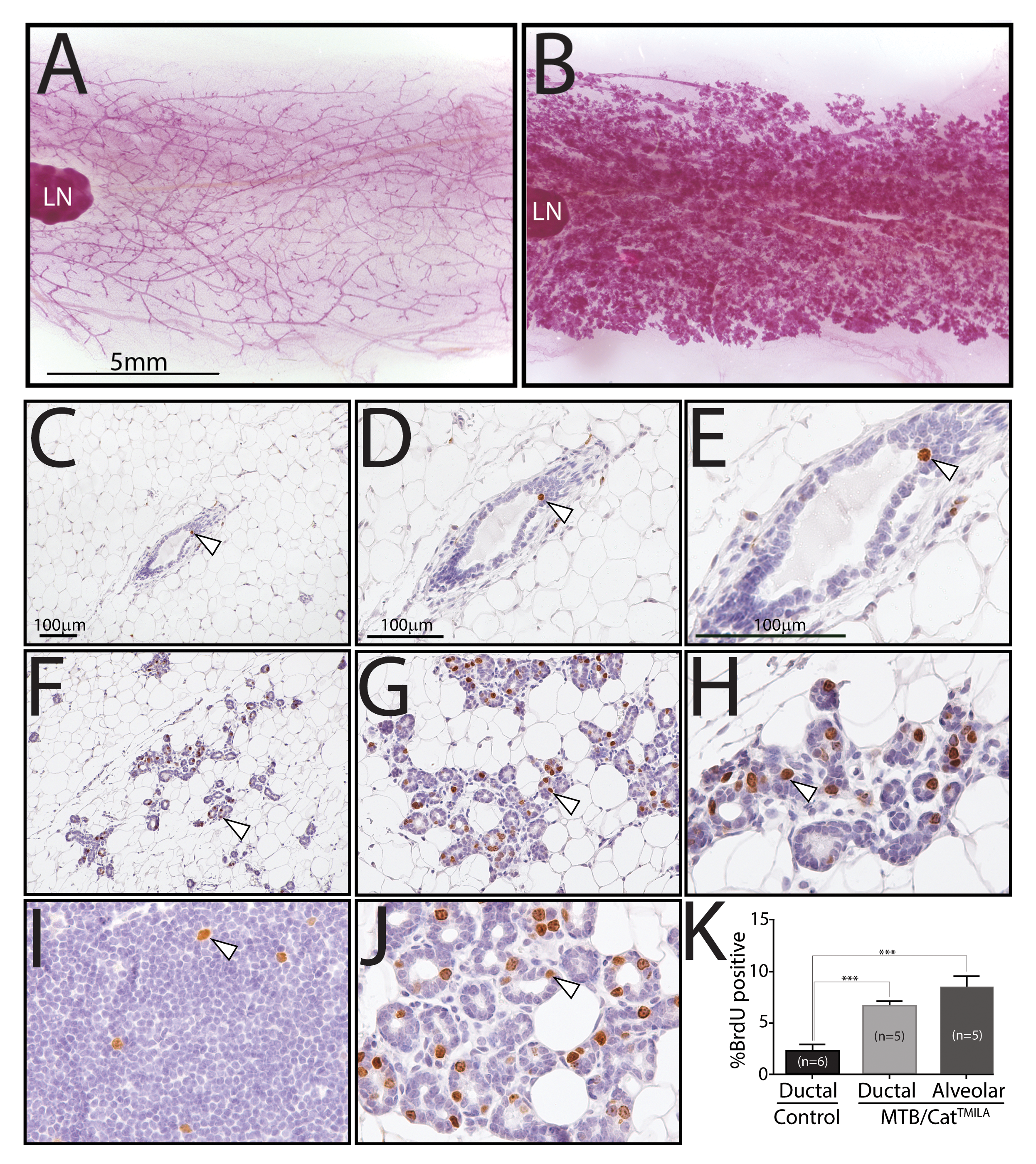

Supplement: S1 Fig — (A) and (B) represent low magnification whole mount images of mammary gland tissue isolated from a monogenic control (G6719) and a MTB/CatTMILA bigenic (G6718) respectively following doxycycline administration for 1-week (see Fig 2). Scale bar in (A) applies to (B); LN denotes lymph node in inguinal mammary gland. At increasing magnification, (C-E) show a typical transverse section of a doxycycline-treated control monogenic mammary duct with a single luminal epithelial cell scoring positive for BrdU incorporation (white arrowhead). At increasing magnification, (F-H) show numerous alveolar epithelial cells in the doxycycline-treated MTB/CatTMILA bigenic mammary gland that are immunopositive for BrdU incorporation (white arrowhead). As a positive control for BrdU immunostaining, (I) shows BrdU positive cells in the lymph node of inguinal (#4) mammary gland of the doxycycline-treated monogenic control (white arrowhead). (J) shows the extensive alveologenesis and cellular proliferation (white arrowhead) that occurs in the doxycycline-treated MTB/CatTMILA bigenic mammary gland. Scale bar in (C-E) applies to (F-H) respectively; scale bar in (E) also applies to (I-J). (K) displays a histogram of the mean percentage of cells (± s.e.m.) scoring positive for BrdU incorporation in ductal epithelium of the doxycycline-treated control monogenic (n = 6) and in the ductal and alveolar epithelium of the doxycycline-treated MTB/CatTMILA bigenic sibling (n = 5); ***denotes P value <0.0001. (TIF) [file pone.0173014.s001.tif]

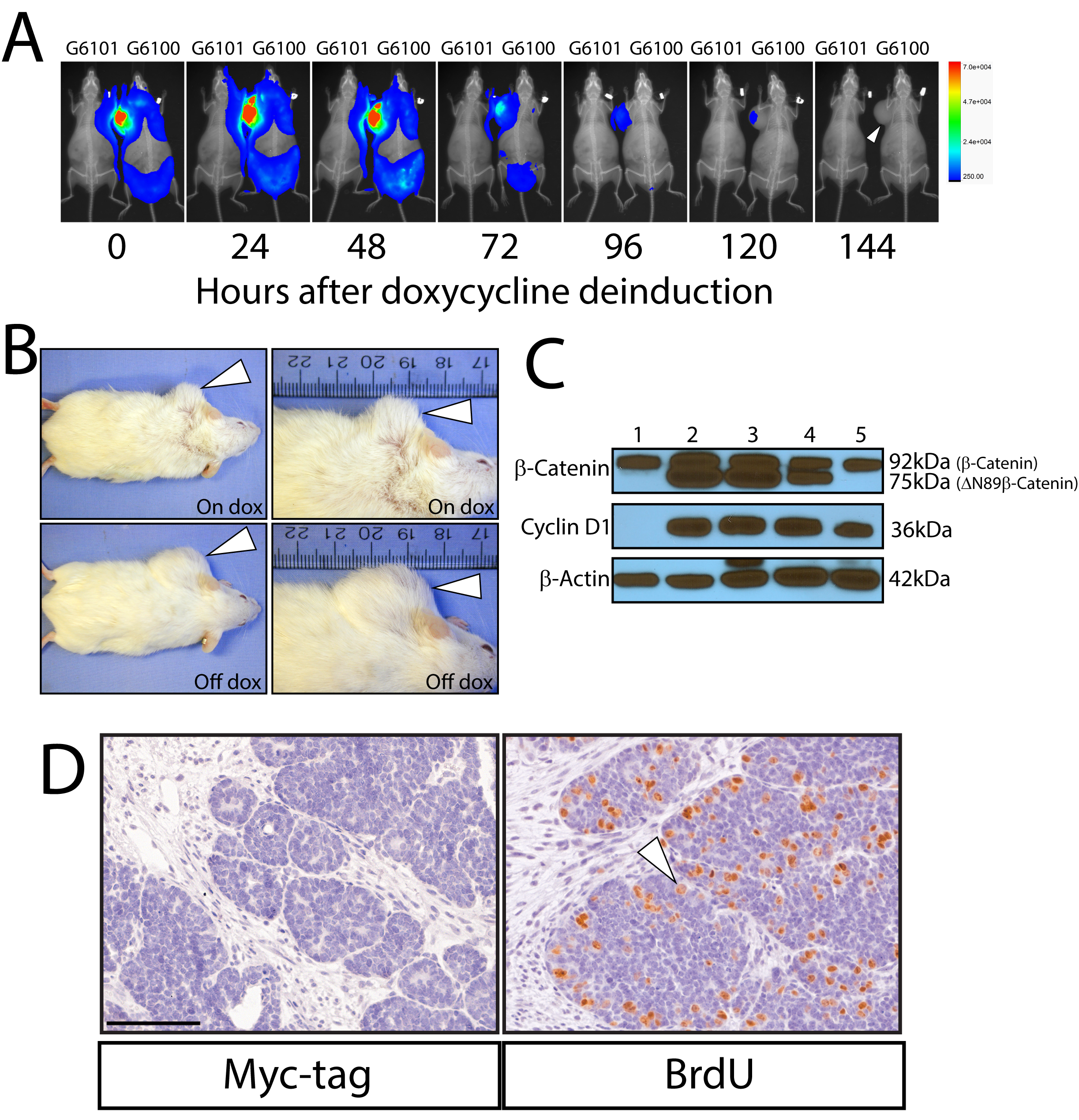

Supplement: S2 Fig — (A) Overlay of whole body bioluminescence and x-ray images of monogenic control (G6101) and MTB/CatTMILA (G6100) bigenic following removal of doxycycline for the time period indicated. By 120 hours post doxycycline removal, transgene-derived luciferase activity is significantly attenuated but mammary tumor mass is not reduced (white arrowhead). (B) Top two panels (low and high magnification) show MTB/CatTMILA (G6100) bigenic at 0h after doxycycline withdrawal; white arrowhead points to thoracic mammary gland tumor shown in (A). Two bottom panels (low and high magnification) show the MTB/CatTMILA (G6100) bigenic 144 hours following doxycycline withdrawal. Note that the mammary tumor has not decreased in size following de-induction of the transgene (white arrowhead). (C) Western immunoblot of protein isolated from: WT mammary epithelial cells (lane 1), three separate MTB/CatTMILA bigenic mammary tumors on doxycycline (lanes 2–4), and MTB/CatTMILA bigenic mammary tumor off doxycycline for 144 days (lane 5). Note the absence of transgene-derived ΔN89β-catenin protein in lane 5. High levels of cyclin D1 protein expression is detected in all mammary tumor samples (lanes 2–5); β-actin serves as a loading control. (D) Immunohistochemistry does not detect transgene-derived myc-tagged ΔN89β-catenin in mammary tumor tissue (adenocarcinoma) derived from MTB/CatTMILA bigenic that are off doxycycline for 144 hours. The same mammary tumor tissue is highly proliferative as evidenced by numerous tumor cells scoring positive for BrdU incorporation (white arrowhead). Scale bar applies to both panels. (TIF) [file pone.0173014.s002.tif]

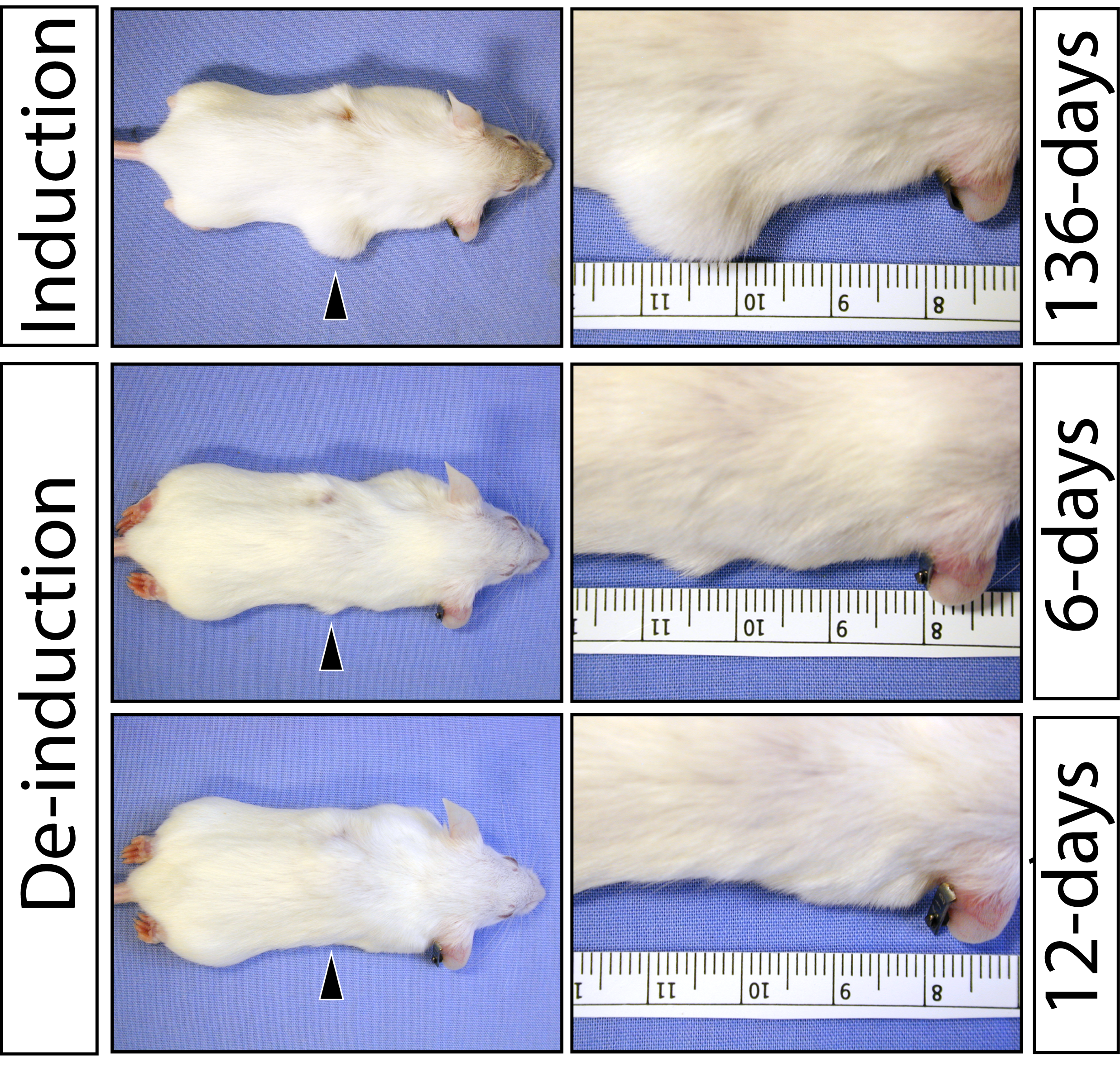

Supplement: S3 Fig — A representative MTB/CatTMILA bigenic with a palpable mammary tumor is shown (black arrowhead) in the top two panels (low and high magnification). For this representative mouse, 136 days of doxycycline administration was required to induce a palpable thoracic (#3) mammary tumor with a ~1cm diameter. Following 6-days on a standard diet without doxycycline (or de-induction), the size of the same tumor rapidly reduced (middle two panels). By 12-days without doxycycline in the diet, the thoracic mammary tumor in the MTB/CatTMILA bigenic is undetectable by manual palpation. Of the MTB/CatTMILA bigenic mice in this study (n = 20), 18 mice showed rapid mammary tumor regression within 14-days whereas 2 mice did not show mammary tumor regression within this time period (S2 Fig). (TIF) [file pone.0173014.s003.tif]
